# A Combination of Amide Proton Transfer, Tumor Blood Flow, and Apparent Diffusion Coefficient Histogram Analysis Is Useful for Differentiating Malignant from Benign Intracranial Tumors in Young Patients: A Preliminary Study

**DOI:** 10.3390/diagnostics14121236

**Published:** 2024-06-12

**Authors:** Fumine Tanaka, Masayuki Maeda, Ryohei Nakayama, Katsuhiro Inoue, Seiya Kishi, Ryota Kogue, Maki Umino, Yotaro Kitano, Makoto Obara, Hajime Sakuma

**Affiliations:** 1 Department of Radiology, Mie University School of Medicine, 2-174 Edobashi, Tsu 5148507, Mie, Japan; 2 Department of Neuroradiology, Mie University School of Medicine, 2-174 Edobashi, Tsu 5148507, Mie, Japan; 3 Department of Electronic and Computer Engineering, Ritsumeikan University, 1-1-1 Noji-higashi, Kusatsu 5250058, Shiga, Japan; 4 Department of Radiology, Mie University Hospital, 2-174 Edobashi, Tsu 5148507, Mie, Japan; 5 Department of Neurosurgery, Mie University School of Medicine, 2-174 Edobashi, Tsu 5148507, Mie, Japan; 6 MR Clinical Science, Philips Japan, 2-13-37 Konan, Minato 1088507, Tokyo, Japan

**Keywords:** amide proton transfer, pseudocontinuous arterial spin labeling, apparent diffusion coefficient, pediatric brain tumor, histogram analysis

## Abstract

Purpose: To evaluate the amide proton transfer (APT), tumor blood flow (TBF), and apparent diffusion coefficient (ADC) combined diagnostic value for differentiating intracranial malignant tumors (MTs) from benign tumors (BTs) in young patients, as defined by the 2021 World Health Organization classification of central nervous system tumors. Methods: Fifteen patients with intracranial MTs and 10 patients with BTs aged 0–30 years underwent MRI with APT, pseudocontinuous arterial spin labeling (pCASL), and diffusion-weighted imaging. All tumors were evaluated through the use of histogram analysis and the Mann–Whitney U test to compare 10 parameters for each sequence between the groups. The diagnostic performance was evaluated using receiver operating characteristic (ROC) curve analysis. Results: The APT maximum, mean, 10th, 25th, 50th, 75th, and 90th percentiles were significantly higher in MTs than in BTs; the TBF minimum (min) was significantly lower in MTs than in BTs; TBF kurtosis was significantly higher in MTs than in BTs; the ADC min, 10th, and 25th percentiles were significantly lower in MTs than in BTs (all *p* < 0.05). The APT 50th percentile (0.900), TBF min (0.813), and ADC min (0.900) had the highest area under the curve (AUC) values of the parameters in each sequence. The AUC for the combination of these three parameters was 0.933. Conclusions: The combination of APT, TBF, and ADC evaluated through histogram analysis may be useful for differentiating intracranial MTs from BTs in young patients.

## 1. Introduction

Primary brain tumors in children are the second most common type of tumor behind leukemia, and the incidence of central nervous system (CNS) tumors in the 15–29-year-old age group accounts for 6% of all neoplasms [1]. In contrast to those in adults, the pathological types of CNS tumors in young patients are highly heterogeneous [2,3,4,5,6,7]. In addition, typical extra-axial tumors, including meningiomas, occur less frequently in younger patients [8,9]. Nevertheless, differentiating between intracranial malignant tumors (MT) and benign tumors (BT) is essential because the tumor type influences the treatment strategy and ultimately the prognosis. In 2021, the World Health Organization (WHO) classification of CNS tumors was updated, and an integrated diagnostic approach that includes both the histopathological examination and genetic and molecular status was introduced [10]. Consequently, a re-evaluation of the imaging characteristics of MTs and BTs is recommended.

Magnetic resonance imaging (MRI) plays a crucial role in tumor diagnosis. Multiparametric MRI with different types of scan sequences provides quantitative information about various tumor characteristics, which could contribute to differential diagnoses [11,12,13,14]. These quantifiable imaging data are compatible with objective analysis methods, such as histogram analysis, which are considered reproducible and repeatable when compared to subjective analysis methods, including visual evaluation [11,12,13,14]. Thus, these analyzed data can improve the diagnostic performance, even for brain tumors defined by the 2021 WHO classification. First, the apparent diffusion coefficient (ADC) value obtained through diffusion-weighted imaging (DWI) is a common parameter that reflects the cell density of tumors [15]. ADC values are reportedly useful for predicting the grade of intracranial tumors in children and for distinguishing between different pediatric brain tumor types [9,16,17,18]. In addition, arterial spin labeling (ASL) techniques provide tumor blood flow (TBF) values that can predict either low- or high-grade pediatric brain tumors [19,20,21]. Furthermore, amide proton transfer (APT) imaging provides the amide proton density in tumors, which is reportedly useful in grading gliomas in adults [22,23,24,25]. These imaging techniques, including DWI, ASL, and APT images, do not require contrast agents, which is especially advantageous for young patients. Previous studies have documented gadolinium retention in the brain and bone tissues of patients without severe renal diseases [26,27]. Additionally, there is more concern with the expectedly longer exposure period in pediatric patients than in adult patients, although the clinical meaning of gadolinium retention remains unknown [27]. Therefore, MRI without contrast agents is desirable, especially for young patients. However, we only found one paper concerning the usefulness of APT imaging in the differentiation of pediatric brain tumors [28], and no reports on studies combining APT imaging, ASL imaging, and ADC maps. This study aimed to evaluate the APT, TBF, and ADC combined diagnostic value for differentiating intracranial MTs from BTs in children and young adults.

## 2. Materials and Methods

### 2.1. Subjects

This study was approved by the ethics committee of our university, and the requirement for written informed consent was waived because of the retrospective study design. All procedures were performed according to the principles of the World Medical Association Declaration of Helsinki. We retrospectively identified 64 patients with suspected intracranial tumors who underwent pretreatment MRI between April 2019 and November 2023. Patients were included if they met the following criteria: (a) aged 0–30 years, (b) available pretreatment 3-T MRI, including APT, pCASL, DWI, T1-weighted, contrast-enhanced, and T2-weighted images; (c) primary intracranial tumors proven through resected or biopsy specimens; and (d) a maximum tumor diameter >10 mm. The exclusion criteria were as follows: (1) metastatic brain tumors because this study was limited to primary brain tumors; and (2) typical extra-axial tumors, such as meningioma, solitary fibrous tumor, or schwannoma. We categorized all tumors as MTs or BTs. MTs included grades 3 and 4 or those diagnosed as high-grade gliomas according to the WHO 2021 classification. Tumors with the International Classification of Disease (ICD) behavior code/3 were also classified as MTs if no WHO grade was assigned to the tumors. Conversely, BTs included grades 1 and 2 or tumors diagnosed as low-grade gliomas according to the WHO 2021 classification.

### 2.2. MRI Protocol

All patients underwent 3-T MRI (Ingenia; Philips Medical Systems, Best, The Netherlands) with a 32-channel phased-array head coil. The pulse sequence parameters were as follows: APT image, three-dimensional (3D) turbo spin-echo sequence; pulse-train, saturation pulse duration, 2.0 s; saturation B1 rms, 2.0 μT; duty-cycle DCsat = 100%, 40 × 50 ms elements with sinc-gauss shape; alternated 2 RF channel transmission via the body coil; 7-point APT-w imaging protocol, S0 = −1560 ppm, ±2.7, ±3.5, and ±4.3 ppm; acquisition at +3.5 ppm is acquired 3 times with different echo shifts (0, ±0.5 ms) deriving a 3-point Dixon-type B0 map (water frequency mapping) for B0 correction; lipid suppression = SPIR; repetition time (TR), shortest (6139 ms); echo time (TE), shortest (7.8–8.8 ms); field of view (FOV), 230 × 230 mm^2^; matrix = 128 × 128; slice thickness, 6 mm; resolution, 0.9 × 0.9 × 6 mm; number of slices, 3–10 slices; and scan time, 2 min 51 s to 3 min 47 s.

The APT signal is defined as the asymmetry of the magnetization transfer ratio at 3.5 ppm: MTR_asym_ (3.5 ppm) [23], and is calculated as follows:

$$APT\;signal={MTR}_{asym}\left(3.5\;ppm\right)=[S_{sat}(-3.5\;ppm)-S_{sat}(+3.5\;ppm)]/S_{0}$$

where S_sat_(−3.5 ppm), S_sat_(+3.5 ppm), and S_0_ are the signal intensities obtained at −3.5, +3.5, and 1560 ppm, respectively [22].

The pCASL images were acquired as follows: 3D turbo spin-echo sequence; TR, 6000 ms; TE, 40 ms; FOV, 240 × 240 mm^2^; matrix size, 80 × 80; slice thickness, 3 mm (over contiguous); resolution, 3 × 3 × 3 mm; labeling duration, 1650 ms; postlabeling delay, 2000 ms or 2200 ms; number of slices, 40; and acquisition time, 5 min. The TBF was calculated according to the following equation [19]:

$$TBF=\frac{6000\cdot \lambda \cdot \left({SI}_{control}-{SI}_{label}\right)\cdot e^{\frac{PLD}{T_{1,blood}}}}{2\cdot \alpha \cdot T_{1,blood}\cdot {SI}_{PD}\cdot (1-e^{-\frac{\tau }{T_{1,blood}}})}\;[\mathrm{mL}/100\;g/\min]$$

where λ is the blood/tumor–tissue water partition coefficient (1.0 g/mL), and SI_control_ and SI_label_ are the time-averaged signal intensities in the control and label images, respectively. T_1,blood_ is the longitudinal relaxation time of blood (1650 ms), α is the labeling efficiency (0.85), SI_PD_ is the signal intensity of a proton density-weighted image, and τ is the labeling duration (1650 ms). The value of λ was 1.0 mL/g.

The DWI was performed as follows: sequence, echo planar imaging; FOV, 220; matrix, 112 × 168 (scan% = 150); recon matrix, 224; SENSE, 3.0; slices, 48; TE/TR, 87/5865 (shortest); NSA, 1; measured voxel size = 1.96 × 1.32 × 3.00; reconstructed voxel size, 0.98 × 0.98 × 3.00; and scan time, 1 min 10 s. The other sequences included 3D fluid-attenuated inversion recovery (FLAIR), susceptibility-weighted imaging (SWI), T2-weighted imaging, and 3D T1-weighted imaging with and without gadolinium agents (Supplementary Table S1).

### 2.3. Image Analysis

**Figure 1:**
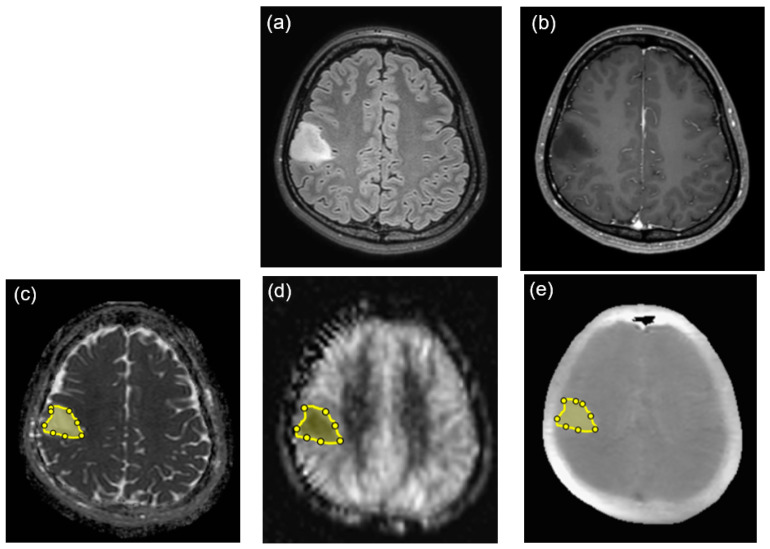
Segmentation of the tumors on the right parietal lobe. The solid portion of the tumor showing a hyperintense signal on FLAIR (**a**) and no enhancement on contrast-enhanced T1WI (**b**) were manually segmented on the ADC map ((**c**), yellow). The segmented region of interest was copied from the ADC map of the software to the pCASL image ((**d**), yellow) and APT image ((**e**), yellow) of the software.

A custom software application developed in MATLAB 2020a (MathWorks, Natick, MA, USA) was used to perform image analysis. The custom software displays the APT image, pCASL image, and ADC map for the same patient side by side on a monitor. Two board-certified neuroradiologists (F.T. and R.K.) reviewed the images of all MRI sequences. First, we identified tumors on T1-weighted, T2-weighted, and contrast-enhanced T1-weighted images. Using the software, we manually drew the region of interest (ROI) around the solid part of the tumor margin in the maximum diameters on the ADC map (Figure 1). We drew the ROIs within an entire solid part of a tumor as was visually traced, avoiding areas of necrosis, cyst, or hemorrhage. The segmented ROI was then copied from the ADC map and pasted onto the APT and pCASL images via the software. Histogram analysis was performed to determine histogram features for each image or map. The following 10 objective features were determined as histogram parameters in the custom software: (1) minimum (min), (2) mean, (3) maximum (max), (4) 10th percentile, (5) 25th percentile, (6) 50th percentile, (7) 75th percentile, (8) 90th percentile, (9) skewness, and (10) kurtosis. The histogram parameters of APT, TBF, and ADC were measured twice in each ROI and averaged.

### 2.4. Statistical Analysis

SPSS v. 29.0 software (IBM SPSS Statistics for Windows, IBM Corp., Armonk, NY, USA) was used to perform statistical analysis. Pearson’s chi-square test was performed to compare the sex and diagnostic methods between the two groups, and the Mann–Whitney U test was performed to compare the age and tumor maximum diameter between the groups. Interobserver agreement between two readers for TBF, ADC, and APT values was assessed using the intraclass correlation coefficient (ICC) (2,k). ICC values < 0.50, 0.50–0.75, 0.75–0.90, and >0.90 indicated poor, moderate, good, and excellent reliability, respectively [29]. All 10 parameters of the APT, TBF, and ADC values were evaluated. The Mann–Whitney U test was performed to determine significant differences between groups, followed by the Shapiro–Wilk test to assess the normality of the data distribution. Values of *p* < 0.05 were accepted as indicating statistical significance. Receiver operating characteristic (ROC) curve analyses were performed to investigate the diagnostic performance of the APT, TBF, and ADC. Binomial logistic regression was performed to combine all optimal parameters for each sequence. We considered area under the curve (AUC) values < 0.7, 0.7–0.9, and >0.9 to indicate low, medium, and high diagnostic performances, respectively. The maximum of the Youden index (Youden index = sensitivity + specificity − 1) was used to calculate the cutoff values. Values of *p* < 0.05 were accepted as indicating statistical significance.

## 3. Results

### 3.1. Patients

**Figure 2:**
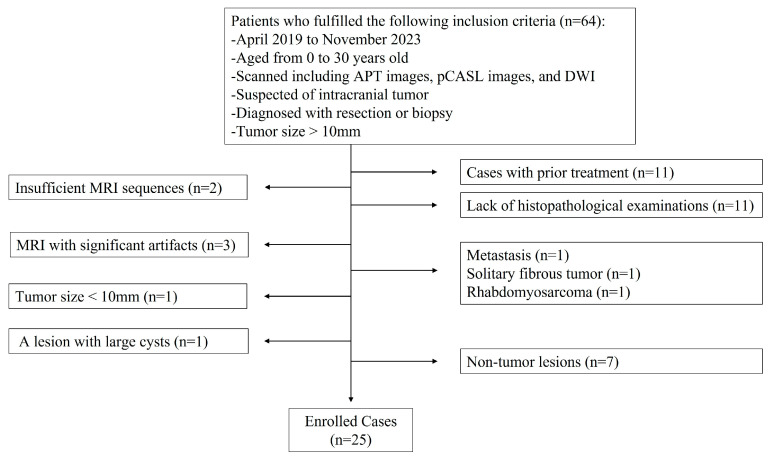
The patient selection diagram. Abbreviations: APT, amide proton transfer; pCASL, pseudocontinuous spin labeling; DWI, diffusion weighted image.

A total of 25 patients included 15 with MTs (10 males and 5 females; age range, 1 month–29 years old; mean age, 10.87 ± 9.17 years old) and 10 with BTs (5 males and 5 females; age range, 1–30 years old; mean age, 14.30 ± 11.44 years old). Figure 2 illustrates the patient selection. The patient characteristics are presented in Table 1. The sex, age, maximum tumor diameter, and diagnostic methods were not significantly different between the patients with MTs and those with BTs (*p* = 0.405, 0.415, 0.061, and 0.096, respectively). The histopathological and molecular types of tumors are described in Table 2.

### 3.2. Interobserver Agreement

ICCs and 95% confidence intervals for each parameter are shown in Supplementary Table S2. All APT parameters except for APT min and skewness, TBF mean, 25th, 50th, and 75th percentiles, and all ADC parameters except for ADC max, skewness, and kurtosis showed excellent reliability. The APT min, TBF max, 10th, and 90th percentile, and skewness, as well as the ADC max, skewness, and kurtosis showed good reliability. The APT skewness, TBF min, and kurtosis had moderate reliability.

### 3.3. Comparisons of APT, TBF, and ADC Parameters between MTs and BTs

**Figure 3:**
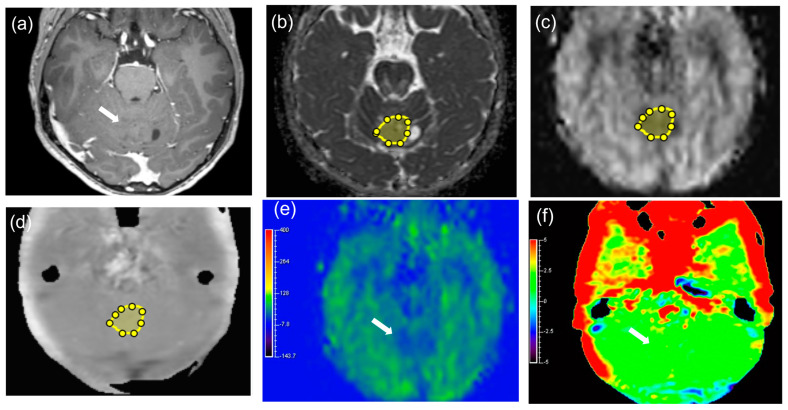

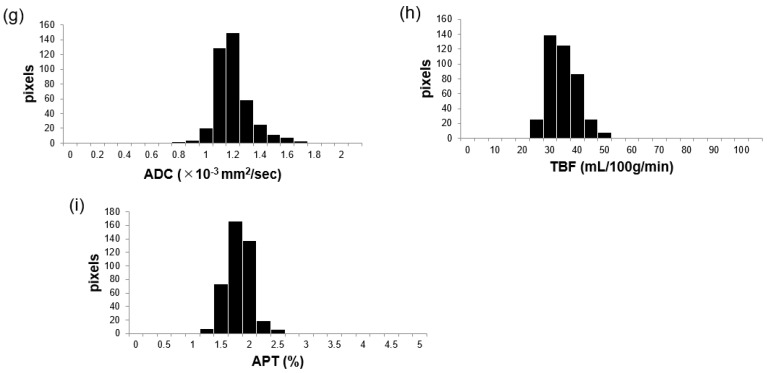
A 10−year−old female with pilocytic astrocytoma in the cerebellar vermis as a representative case of BT, patient number 25 (arrows). Contrast−enhanced T1WI (**a**) showed no contrast enhancement. The ROI was manually drawn on the ADC map ((**b**), yellow), and the ROI was copied from the ADC map to both the pCASL grayscale image ((**c**), yellow) and APT grayscale image ((**d**), yellow). The ADC map (**b**) showed a slightly high ADC value (ADC mean = 1.15 × 10^−3^ mm^2^/s, ADC min = 0.68 × 10^−3^ mm^2^/s). The pCASL color image (**e**) revealed a low TBF mean value and high TBF min value (TBF mean = 32.26 mL/100 g/min, TBF min = 22.65 mL/100 g/min). The APT color image (**f**) showed no apparent increase in the APT signal (APT mean = 1.68%, APT 50th percentile = 1.69%). The ADC histogram (**g**), TBF histogram (**h**), and APT histogram (**i**) are shown.

**Figure 4:**
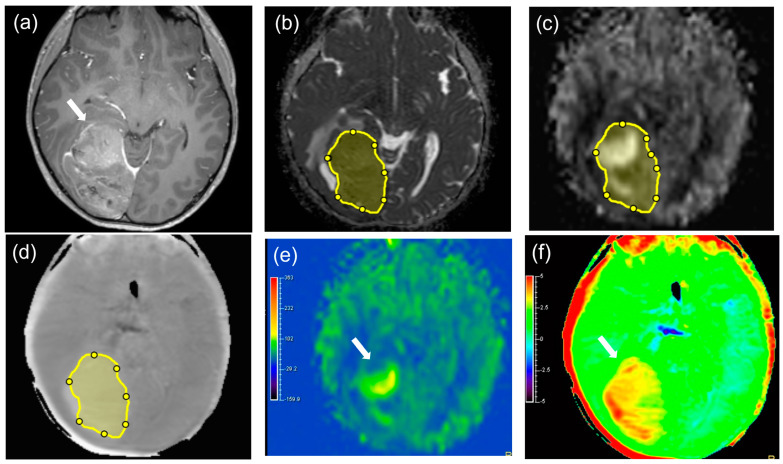

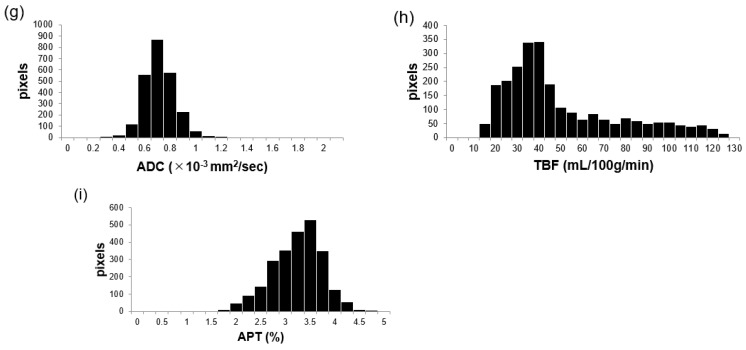
A 9−year−old male with paediatric−type diffuse high−grade glioma; NOS in the right occipital lobe as a representative case of MT, patient number 5 (arrows). Contrast−enhanced T1WI (**a**) showed mild contrast enhancement. The ROI was manually drawn on the ADC map ((**b**), yellow), and the ROI was copied from the ADC map to both the pCASL grayscale image ((**c**), yellow) and APT grayscale image ((**d**), yellow). The ADC map (**b**) showed a low ADC value (ADC mean = 0.67× 10^−3^ mm^2^/s, ADC min = 0.17 × 10^−3^ mm^2^/s). The pCASL color image (**e**) revealed a high TBF mean value and a low TBF min value (TBF mean = 47.70 mL/100 g/min, TBF min = 9.95 mL/100 g/min). The APT color image (**f**) showed a high APT value (APT mean = 3.11%, APT 50th percentile = 3.16%). The ADC histogram (**g**), TBF histogram (**h**), and APT histogram (**i**) are shown.

Figure 3 and Figure 4 show representative cases of MT and BT, respectively. Supplementary Table S3 shows the results of the Shapiro–Wilk test for each parameter. Table 3, Supplementary Table S4 shows the parameter measurements of the APT, TBF, and ADC in MTs and BTs, respectively. There were significant differences in the APT max, mean, 10th, 25th, 50th, 75th, and 90th percentiles (*p* = 0.004, <0.001, 0.008, 0.003, <0.001, 0.003, and 0.010, respectively), TBF min and kurtosis (*p* = 0.008 and 0.026, respectively), and ADC min, 10th, and 25th percentiles (*p* < 0.001, *p* = 0.005, and 0.023, respectively) between MTs and BTs. The median values of the APT max, mean, 10th, 25th, 50th, 75th, and 90th percentiles were higher in MTs (5.23, 3.27, 2.27, 2.85, 3.26, 3.72, and 4.03%, respectively) than in BTs (2.43, 1.74, 1.21, 1.41, 1.76, 1.87, and 2.20%, respectively). The median value of the TBF min in MTs (3.22 mL/100 g/min) was lower than in BTs (9.46 mL/100 g/min). The median value of TBF kurtosis in MTs (3.76) was higher than in BTs (2.52). The median value of the TBF max in MTs (45.93 mL/100 g/min) was higher than in BTs (41.06 mL/100 g/min), although it was not statistically significant. The median values of the ADC min, 10th, and 25th percentiles in MTs (0.41, 0.66, and 0.82 × 10^−3^ mm^2^/s, respectively) were lower than in BTs (0.83, 0.96, and 1.01 × 10^−3^ mm^2^/s).

Supplementary Figure S1 shows the scatter plots of the APT 50th percentile, TBF min, TBF max, and ADC min for each tumor.

### 3.4. Diagnostic Performance for Differentiating MTs from BTs

**Figure 5:**
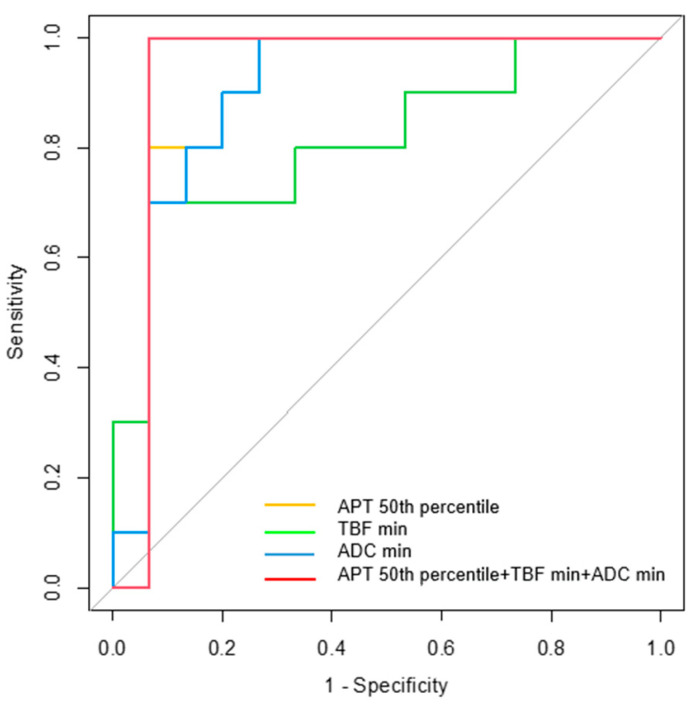
ROC curve analyses for differentiating malignant tumors from benign tumors. The areas under the curves (AUC) for the APT 50th percentile (AUC = 0.900), TBF min (AUC = 0.813), and ADC min (AUC = 0.900) indicate medium diagnostic performances. The AUC for the combination of the APT mean, APT 10th, APT 25th, and APT 50th percentile, TBF max, and ADC min indicates a high diagnostic performance (AUC = 0.933).

Supplementary Table S5 shows the diagnostic performance of all parameters. In differentiating MTs from BTs, the APT 50th percentile, TBF min, and ADC min had the highest AUC of any sequence parameter (0.900, 0.813, and 0.900, respectively). Table 4 and Figure 5 summarize the diagnostic performance of these parameters alone and in combination. The AUCs for the APT 50th percentile (0.900; 95% confidence interval (CI), 0.764–1.000, *p* = 0.001) and TBF min (0.813; 95% CI, 0.632–0.995, *p* = 0.009) indicate a moderate diagnostic performance, and the ADC min (0.900; 95% CI, 0.771–1.000, *p* = 0.001) indicates a high diagnostic performance. The AUC for the combination of the APT 50th percentile, TBF min, and ADC min indicates a high diagnostic performance (0.933; 95% CI, 0.807–1.000, *p* < 0.001).

## 4. Discussion

In this study, the diagnostic performance of the combination of ADC, TBF, and APT values for differentiating between MTs and BTs in young patients was improved when compared to that of each parameter alone. Thus, we have shown that multiparametric MRI may be applicable to a wider range of brain tumors in young patients in the differential diagnosis of MTs and BTs. In addition, we performed a histogram analysis to obtain more detailed information about brain tumor heterogeneity. This is the first study to evaluate the usefulness of combining the APT, pCASL, and ADC images via histogram analysis for differentiating intracranial MTs from BTs, including both gliomas and non-gliomas in young patients.

In our study, the APT image showed a better diagnostic performance than the pCASL image and the same diagnostic performance as the ADC map. Togao et al. and Wen et al. showed that the APT signal intensity indicated different mobile protein and peptide concentrations in tumors, and was positively correlated with the glioma grade and cell density [24,30]. Park et al. and Nakajo et al. suggested that the amide protons in endogenous mobile proteins and peptides located in the cytoplasm may be the main source of APT signal intensity [31,32]. Additionally, mobile proteins within microscopic necrotic foci, fluid accumulation within vesicles, or extracellular proteins and peptides may also contribute to increased APT signals [24,32]. In the APT histogram analysis, the APT 50th percentile showed the best AUC value of all parameters, followed by the APT mean. The AUC value was higher for the APT mean than for the APT 90th percentile, which is consistent with previous studies [25,33,34]. On the other hand, Su et al. found that higher percentiles of the APT parameter, such as the 95th percentile, were more important than lower percentiles in differentiating between high-grade and low-grade gliomas [35]. This is inconsistent with our results, possibly because of the variety of tumor types, including non-gliomas, in our study. Histogram analysis metrics, such as percentiles, kurtosis, and skewness, are strong and reliable quantitative surrogate markers of tumor heterogeneity [12,36]. Therefore, we believe that tumor microenvironments may be masked when evaluating only a single parameter, such as the mean value [12]. According to our results, the APT 50th percentile may provide a more accurate description of tumor microenvironments than the max or mean values when evaluating both gliomas and non-gliomas in young patients.

The TBF min achieved the best diagnostic performance among all TBF parameters, and the TBF min values in MTs were significantly lower than in BTs. To the best of our knowledge, there is no literature demonstrating that the TBF min is useful in differentiating MTs from BTs. These results may be due to the necrosis of MTs, suggesting that the TBF min in MTs corresponds to necrosis. However, necrosis is essentially absent in BTs. Therefore, it seems that BTs have a higher TBF min than MTs. On the other hand, all TBF parameters, except for the min and kurtosis, did not show significant differences between MTs and BTs. Kang et al. showed that there was no significant difference in the 90th percentile and mean relative TBF derived through pCASL between low- and high-grade gliomas, which is in line with our study [33]. In contrast, two reports showed that the maximum or mean TBF of high-grade tumors was significantly higher than that of low-grade tumors when using pCASL imaging [20,21]. Thus, evaluating the TBF alone may be less reliable than evaluating it in conjunction with other measures, such as the ADC or APT. According to our ROC analysis, the TBF min showed the best diagnostic performance among all TBF parameters. The most plausible explanation for this could be the diversity of tumor types in our patients. Furthermore, because the WHO 2021 classification intrinsically introduces molecular diagnostic methods, vascular features may not be critical for tumor diagnosis. The usefulness of the perfusion findings in the diagnosis of H3K27-altered diffuse midline glioma (DMG) is limited by the histopathological variation from low- to high-grade astrocytic tumors, and no clear conclusion has been reached [37,38]. In our study, three patients diagnosed with H3K27-altered DMG showed variable TBF values due to different histopathological features, such as low-grade astrocytoma and glioblastoma.

Sugahara et al. reported that the ADC value may be mainly influenced by the tumor cellularity, and the ADC min value correlated well with the histological cellularity and glioma grade [39]. Although their study used ROI placement via visual assessment rather than histogram analysis, their results are consistent with our results, showing that the ADC min has the best diagnostic performance when ADC histogram analysis is used. Thus, our study using histogram analysis confirmed that the ADC min is also useful in differentiating MTs and BTs in young patients with heterogeneous tumor types.

Previously, two reports have described that the combination of the APT, pCASL-derived TBF, and ADC improved the diagnostic performance of the astrocytic tumor grade when compared to each parameter alone [25,33]. However, only a few histogram parameters were used in these studies, such as the ADC 10th or 20th percentile, TBF 90th percentile, and APT 90th percentile [25,33]. We showed optimal diagnostic performance parameters from a wider range of histogram parameters than previous reports, and we demonstrated the potential of histogram analysis in differentiating MTs and BTs.

This study had several limitations. First, this study is preliminary because the number of patients, especially those with BT, was small. Further studies with a larger number of young patients are needed to confirm the efficacy and reliability of APT, pCASL, and ADC maps in the evaluation of intracranial tumors. In addition, a larger dataset would prevent overfitting cut-off values of the diagnostic performance. In the parameter comparison analysis between MTs and BTs, *p* value correction was not performed. Thus, the correction is necessary for future studies. We did not evaluate the entire slice of each scan sequence for each tumor. However, single-slice evaluation is practical in clinical practice. Furthermore, Sakata et al. reported that there were no significant differences in the glioma grade between single-slice APT analysis and whole-brain analysis [40]. Finally, we did not investigate the correlations between the ADC, TBF, and APT signal values and the histopathological findings, such as the cell density, microscopic cysts, necrosis, hemorrhage, or the microvessel density. It would be useful to investigate these correlations in a future study to determine the importance of each imaging modality in relation to histopathology. The improved study design can enhance the diagnostic performance in brain tumors, which can be caused by genetic factors, obesity, or any other factors [41].

## 5. Conclusions

The APT, TBF, and ADC combination evaluated through histogram analysis was useful for differentiating intracranial MTs from BTs in children and young adults.

## Figures and Tables

**Table 1:** Patient’s characteristics.

	MTs (n = 15)	BTs (n = 10)	*p*-Value
Sex			0.405
Male: Female	10:5	5:5	
Age			0.415
Range (years old)	0–29	1–30	
Mean age (years old)	10.87 ± 9.17	14.30 ± 11.44	
Tumor maximum diameter			0.061
Range (mm)	28–83	15–87	
Mean (mm)	50.67 ± 16.49	35.40 ± 22.32	
Diagnostic method			0.096
Resection: Biopsy	11:4	5:5	

Abbreviations: MT, malignant tumor; BT, benign tumor.

**Table 2:** Patient’s histopathological and molecular types of tumors.

Tumor Type	Patient Number	Age	Sex	Location	Diameter (mm)	Diagnostic Method	Diagnosis and WHO2021 Grade or ICD Behavior Code	Histopathological Diagnosis and Molecular Status
M	1	3	F	Lt. cerebellopontine angle	58	70% resection	Diffuse midline glioma H3 K27-altered, grade4	Glioblastoma Histone H3 K27M mutation was detected.
M	2	6	M	Bilateral thalamus	34	Biopsy	Diffuse midline glioma H3 K27-altered, grade4	Diffuse astrocytoma without microvascular proliferation or necrosis Histone H3 K27M mutation was detected.
M	3	29	M	Bilateral thalamus~midbrain~cerebellum	81	20% resection	Diffuse midline glioma H3 K27-altered, grade4	Diffuse astrocytoma without microvascular proliferation or necrosis Histone H3 K27M mutation was detected.
M	4	1	F	Lt. lateral ventricle~frontal lobe	83	20% resection	Astroblastoma, *MN1*-altered, ICD/3	Astroblastoma, high-grade, with *MN1* alteration
M	5	9	M	Rt. occipital lobe	68	80% resection	Paediatric-type diffuse high-grade glioma, NOS	Glioblastoma
M	6	12	F	Rt. frontal lobe	50	100% resection	Paediatric-type diffuse high-grade glioma, H3-wildtype and IDH-wildtype, grade4	Glioblastoma
M	7	17	M	Fourth ventricle	44	70% resection	Medulloblastoma, non-WNT/non-SHH, grade 4	Medulloblastoma
M	8	5	M	Fourth ventricle	49	100% resection	Medulloblastoma, NOS, grade 4	Medulloblastoma
M	9	3	F	Rt. lateral ventricle	58	100% resection	Atypical teratoid/rhabdoid tumor, grade 4	Atypical teratoid/rhabdoid tumor
M	10	11	F	Lt. basal ganglia	50	80% resection	Germinoma, ICD/3	Germinoma
M	11	22	M	Pineal region	28	100% resection	Germinoma, ICD/3	Germinoma
M	12	27	M	Pineal region	33	Biopsy	Germinoma, ICD/3	Germinoma
M	13	0 (1 month)	M	Fourth ventricle	45	90% resection	Immature teratoma, ICD/3	Immature teratoma,
M	14	6	M	Pineal region	39	Biopsy	Immature teratoma, ICD/3	Immature teratoma
M	15	12	M	Suprasellar region	40	Biopsy	Mixed germ cell tumor, ICD/3	germinoma and immature teratoma
B	16	30	F	Rt. parietal lobe	36	Biopsy	Astrocytoma, IDH-mutant, grade 2	Diffuse astrocytoma without microvascular proliferation, or necrosis Homozygous deletion of *CDKN2A/B* was not detected.
B	17	22	M	Lt. thalamus~midbrain	19	60% resection	Diffuse low-grade astrocytoma, NOS	Diffuse astrocytoma, IDH-wildtype without microvascular proliferation, or necrosis No *TERT* promoter mutation, *EGFR* gene amplification, or +7/−10 chromosome copy-number alterations were detected.
B	18	30	F	Lt. thalamus~hypothalamus	15	Biopsy	Diffuse low-grade astrocytoma, NOS	Diffuse astrocytoma, IDH-wildtype without microvascular proliferation, or necrosis No *TERT* promoter mutation, *EGFR* gene amplification, or +7/−10 chromosome copy-number alterations were detected.
B	19	3	M	Lt. temporal lobe	23	100% resection	Diffuse low-grade astrocytoma, NOS	Diffuse astrocytoma IDH wildtype without microvascular proliferation, or necrosis *BRAF* p.V600E mutation was detected.
B	20	6	F	Lt. thalamus~midbrain	27	Biopsy	Diffuse low-grade astrocytoma, NOS	Diffuse astrocytoma without microvascular proliferation, or necrosis Histone H3 K27M mutation was not detected.
B	21	6	F	Lt. frontal lobe	52	80% resection	Angiocentric glioma, grade 1	Angiocentric glioma
B	22	26	M	Rt. thalamus~midbrain	18	Biopsy	Pilocytic astrocytoma, grade 1	Pilocytic astrocytoma
B	23	1	M	Fourth ventricle	51	70% resection	Posterior fossa ependymoma, group A, grade 2	Ependymoma
B	24	9	M	Lt. lateral ventricle	87	70% resection	Supratentorial ependymoma, NOS, grade 2	Ependymoma
B	25	10	F	Cerebellar vermis	26	Biopsy	Pilocytic astrocytoma, grade 1	Pilocytic astrocytoma

**Table 3:** Measurements of the APT, TBF, and ADC in MTs and BTs.

	MTs	BTs	*p*-Value
APT max	5.23 (2.52–10.09)	2.43 (1.91–10.12)	0.004 *
APT min	0.87 (−1.41–3.22)	0.81 (−9.09–1.26)	0.807
APT mean	3.27 (0.64–5.76)	1.74 (1.15–2.55)	0.001 *
APT 10th percentile	2.27 (−0.03–4.08)	1.21 (−1.13–1.80)	0.008 *
APT 25th percentile	2.85 (0.20–4.41)	1.41 (0.66–2.23)	0.003 *
APT 50th percentile	3.26 (0.57–5.91)	1.76 (1.07–2.65)	<0.001 *
APT 75th percentile	3.72 (1.06–7.83)	1.87 (1.33–4.11)	0.003 *
APT 90th percentile	4.03 (1.40–9.57)	2.20 (1.78–7.18)	0.010 *
APT skewness	−0.17 (−0.70–0.57)	−0.07 (−0.84–1.07)	0.978
APT kurtosis	2.93 (2.05–6.97)	3.02 (2.43–3.99)	0.683
TBF max	45.93 (9.37–87.99)	41.06 (33.01–64.10)	0.807
TBF min	3.22 (−2.92–19.35)	9.46 (0.64–38.02)	0.008 *
TBF mean	23.44 (1.77–51.46)	29.09 (13.32–47.65)	0.196
TBF 10th percentile	11.76 (−0.16–30.42)	17.58 (5.09–40.37)	0.062
TBF 25th percentile	16.70 (0.15–40.37)	23.12 (10.34–43.10)	0.144
TBF 50th percentile	22.81 (1.19–51.04)	28.49 (12.32–46.99)	0.216
TBF 75th percentile	29.81 (2.91–63.41)	32.59 (15.23–52.36)	0.285
TBF 90th percentile	31.95 (4.60–73.24)	36.54 (19.51–56.07)	0.428
TBF skewness	0.30 (−0.68–1.45)	0.15 (−0.46–1.37)	0.892
TBF kurtosis	3.76 (2.18–5.33)	2.52 (1.98–5.68)	0.026 *
ADC max	2.02 (1.05–3.43)	1.68 (1.18–3.16)	0.261
ADC min	0.41 (0.16–1.27)	0.83 (0.52–1.57)	<0.001 *
ADC mean	0.98 (0.43–1.72)	1.13 (0.96–1.93)	0.160
ADC 10th percentile	0.66 (0.31–1.57)	0.96 (0.71–1.75)	0.005 *
ADC 25th percentile	0.82 (0.35–1.68)	1.01 (0.77–1.86)	0.023 *
ADC 50th percentile	0.93 (0.39–1.74)	1.09 (0.86–1.94)	0.216
ADC 75th percentile	1.08 (0.44–1.92)	1.22 (0.99–2.01)	0.238
ADC 90th percentile	1.30 (0.52–2.49)	1.40 (1.13–2.07)	0.367
ADC skewness	1.22 (−1.27–4.20)	0.20 (−1.11–1.93)	0.103
ADC kurtosis	4.88 (1.65–23.78)	3.18 (1.93–8.19)	0.071

Abbreviations: MT, malignant tumor; BT, benign tumor; APT, amide proton transfer; TBF, tumor blood flow; ADC, apparent diffusion coefficient; Max, maximum; Min, minimum. Data are reported as median and range; the unit for all parameters except for skewness and kurtosis is % for APT, mL/100 g/min for TBF, and 10^−3^ mm^2^/s for ADC. * *p* value < 0.05.

**Table 4:** The highest area under the curve values of each sequence for differentiating MTs from BTs.

Parameters	AUC	95% CI	*p*-Value	Cutoff Value	Sensitivity (%)	Specificity (%)
APT 50th percentile	0.900	0.764–1.000	0.001 *	1.94	93.3	80.0
TBF min	0.813	0.632–0.995	0.009 *	7.35	70.0	93.3
ADC min	0.900	0.771–1.000	0.001 *	0.51	100.0	73.3
APT 50th + TBF min + ADC min	0.933	0.807–1.000	<0.001 *	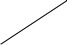	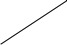	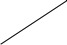

Abbreviation: APT, amide proton transfer (%); TBF, tumor blood flow (mL/100 g/min); ADC, apparent diffusion coefficient (10^−3^ mm^2^/s); min, minimum; AUC, area under the curve; CI, confidence interval. * *p* value < 0.05.

## Data Availability

Data are available in a publicly accessible repository here: http://hdl.handle.net/10076/0002000809 (accessed on 7 June 2024).

## Supplementary Materials

The following supporting information can be downloaded at https://www.mdpi.com/article/10.3390/diagnostics14121236/s1, Figure S1: the scatter plots of the APT 50th percentile, TBF min, TBF max, and ADC min for each tumor; Table S1: Conventional MRI protocol; Table S2: Intraclass correlation coefficients; Table S3: Shapiro–Wilk test for each parameter; Table S4: Receiver operating characteristic curve analysis of all the parameters for differentiating MTs from BTs; Table S5: Receiver operating characteristic curve analysis of all the parameters for differentiating MTs from BTs.
