# Supplementary material for: A Combination of Amide Proton Transfer, Tumor Blood Flow, and Apparent Diffusion Coefficient Histogram Analysis Is Useful for Differentiating Malignant from Benign Intracranial Tumors in Young Patients: A Preliminary Study
Source: Diagnostics (Basel). 2024 Jun 12;14(12):1236. doi: 10.3390/diagnostics14121236 (PMC11202847; doi:10.3390/diagnostics14121236)
Supplement: Supplementary file 1 [file diagnostics-14-01236-s001.zip › Supplementary_Tables_240531.pdf]

Supplementary Table S1

Conventional MRI protocol

	**3D FLAIR**	**SWI-P**	**T2WI**	**3D T1WI**
**Sequence**	IR	FFE	TSE	TFE
**FOV**	250	230	220	260
**Matrix**	256×185 (scan%=72.1)	384×301 (scan%=78.29)	384×346 (scan%=90.14)	288×288 (scan%=100)
**Recon matrix**	480	768	704	384
**Slices**	350	140	42	200
**Thickness**	1.14	2.0	3.0	0.9
**TE/TR**	368 (shortest)/6000	7.2 (echo space=6.2)/31	90/7760 (shortest)	4.6(shortest)/8.2(shortest)
**NSA**	2	1	2	1
**Matrix size**	0.98×0.98×1.14	0.60×0.60×2.0	0.57×0.60×3.0	0.90×0.90×0.9
**Recon matrix size**	0.52×0.52×0.57	0.30×0.30×1.00	0.31×0.31×3.00	0.68×0.68×0.9
**Scan time**	4 minutes 42 seconds	3 minutes 20 seconds	2 minutes 43 seconds	4 minutes 42 seconds
**Others**	SENSE, 2.6×2.6<br>TI=2000<br>equivalent TE=150<br>refocusing, brain FLAIR<br>T2prep, yes<br>fat suppression, SPIR	CS SENSE, 4.0<br>echoes, 4<br>FA, 17	SENSE, 2.0<br>gap, 0.5<br>FA, 90	CS SENSE, 3.0<br>FA, 10

Supplementary Table S2.

Intraclass correlation coefficients (ICC)

**Parameter**	**ICC (95% CI)**
APT max	0.990 (0.962-0.997)

APT min	0.798 (0.249-0.946)
APT mean	0.971 (0.894-0.992)
APT 10th percentile	0.944 (0.791-0.985)
APT 25th percentile	0.959 (0.847-0.989)
APT 50th percentile	0.976 (0.913-0.994)
APT 75th percentile	0.972 (0.895-0.992)
APT 90th percentile	0.981 (0.930-0.995)
APT skewness	0.685 (-0.171-0.915)
APT kurtosis	0.918 (0.695-0.978)
TBF max	0.766 (0.132-0.937)
TBF min	0.628 (-0.384-0.900)
TBF mean	0.921 (0.707-0.979)
TBF 10th percentile	0.882 (0.562-0.968)
TBF 25th percentile	0.927 (0.729-0.980)
TBF 50th percentile	0.949 (0.811-0.986)
TBF 75th percentile	0.941 (0.782-0.984)
TBF 90th percentile	0.864 (0.495-0.963)
TBF skewness	0.815 (0.314-0.950)
TBF kurtosis	0.641 (-0.332-0.904)
ADC max	0.840 (0.406-0.957)
ADC min	0.982 (0.935-0.995)

ADC mean	0.999 (0.996-1.000)
ADC 10th percentile	0.999 (0.996-1.000)
ADC 25th percentile	0.999 (0.997-1.000)
ADC 50th percentile	1.000 (0.999-1.000)
ADC 75th percentile	0.999 (0.996-1.000)
ADC 90th percentile	0.996 (0.986-0.999)
ADC skewness	0.762 (0.117-0.936)
ADC kurtosis	0.882 (0.563-0.968)

Abbreviations: APT, amide proton transfer; TBF, tumor blood flow; ADC, apparent diffusion coefficient; ICC, intraclass correlation coefficient; CI, confidence interval; max, maximum; min, minimum. * *p* value <0.05

Supplementary Table S3.

Shapiro-Wilk test for each parameter

Parameter	Tumor	Statistics	*p* value
APT max	MT	0.950	0.525
	BT	0.664	<0.001*
APT min	MT	0.964	0.761
	BT	0.501	<0.001*
APT mean	MT	0.976	0.934
	BT	0.944	0.594
APT 10th percentile	MT	0.975	0.924
	BT	0.811	0.020*
APT 25th percentile	MT	0.964	0.757
	BT	0.975	0.931
APT 50th percentile	MT	0.976	0.938
	BT	0.974	0.924
APT 75th percentile	MT	0.902	0.103
	BT	0.850	0.058
	MT	0.853	0.019*

APT 90th percentile	BT	0.658	<0.001*
APT skewness	MT	0.958	0.654
	BT	0.929	0.434
APT kurtosis	MT	0.828	0.009*
	BT	0.965	0.844
TBF max	MT	0.963	0.752
	BT	0.867	0.092
TBF min	MT	0.839	0.012*
	BT	0.894	0.188
TBF mean	MT	0.918	0.177
	BT	0.971	0.897
TBF 10th percentile	MT	0.935	0.328
	BT	0.952	0.694
TBF 25th percentile	MT	0.948	0.490
	BT	0.948	0.643
TBF 50th percentile	MT	0.934	0.312
	BT	0.967	0.857
TBF 75th percentile	MT	0.919	0.188
	BT	0.950	0.672
TBF 90th percentile	MT	0.921	0.200
	BT	0.935	0.495
TBF skewness	MT	0.961	0.715
	BT	0.921	0.363
TBF kurtosis	MT	0.973	0.900
	BT	0.725	0.002*
ADC max	MT	0.975	0.927
	BT	0.845	0.050
ADC min	MT	0.839	0.012*
	BT	0.870	0.101
ADC mean	MT	0.925	0.227
	BT	0.770	0.006*
ADC 10th percentile	MT	0.882	0.050
	BT	0.837	0.041*
	MT	0.919	0.188

ADC 25th percentile	BT	0.837	0.041*
ADC 50th percentile	MT	0.931	0.283
	BT	0.831	0.034*
ADC 75th percentile	MT	0.916	0.170
	BT	0.820	0.025*
ADC 90th percentile	MT	0.931	0.283
	BT	0.897	0.204
ADC skewness	MT	0.973	0.902
	BT	0.944	0.594
ADC kurtosis	MT	0.760	0.001*
	BT	0.832	0.035*

Abbreviations: Max, maximum; Min, minimum; MT, malignant tumor; BT, benign tumor. * p value <0.05

Supplementary Table S4. 1st and 3rd quartiles of measurements of APT, TBF, and ADC in MTs and BTs.

	MTs		BTs	
	1st quartile	3rd quartile	1st quartile	3rd quartile
APT max	4.79	7.12	2.09	3.97
APT min	-0.46	1.66	0.15	1.14
APT mean	2.43	3.97	1.44	2.32
APT 10th percentile	1.47	3.01	0.67	1.56
APT 25th percentile	1.95	3.54	0.93	1.74
APT 50th percentile	2.51	3.99	1.47	1.99
APT 75th percentile	2.79	4.47	1.70	2.78
APT 90th percentile	3.07	5.11	1.81	3.17
APT skewness	-0.47	0.29	-0.72	0.53
APT kurtosis	2.36	4.37	2.64	3.49
TBF max	35.76	66.80	38.51	59.70
TBF min	0.09	5.91	4.57	23.84
TBF mean	17.26	28.29	20.31	37.49
TBF 10th percentile	4.99	19.33	12.50	27.79
TBF 25th percentile	10.37	24.31	15.50	30.66

TBF 50th percentile	16.73	28.01	20.04	38.00
TBF 75th percentile	22.74	33.71	24.31	47.30
TBF 90th percentile	25.65	43.73	28.53	51.48
TBF skewness	-0.15	0.84	-0.02	0.52
TBF kurtosis	2.74	4.04	2.14	3.06
ADC max	1.62	2.59	1.35	1.94
ADC min	0.33	0.62	0.72	0.96
ADC mean	0.68	1.28	1.05	1.34
ADC 10th percentile	0.56	0.92	0.84	1.19
ADC 25th percentile	0.59	1.07	0.92	1.28
ADC 50th percentile	0.64	1.28	1.00	1.37
ADC 75th percentile	0.73	1.51	1.11	1.42
ADC 90th percentile	0.83	1.79	1.15	1.71
ADC skewness	0.51	2.44	-0.40	1.25
ADC kurtosis	3.44	10.78	2.53	5.87

Abbreviations: MT, malignant tumor; BT, benign tumor; APT, amide proton transfer; TBF, tumor blood flow; ADC, apparent diffusion coefficient; Max, maximum; Min, minimum

The unit for all parameters except for skewness and kurtosis is % for APT, mL/100g/min for TBF, and 10^{-3} mm^2 /s for ADC. **p* value < 0.05

Supplementary Table S5. Receiver operating characteristic curve analysis of all the parameters for differentiating MTs from BTs

Parameter	AUC	95% CI	*p* value	Cutoff value	Sensitivity (%)	Specificity (%)
**APT max**	0.840	0.647-1.000	0.005*	4.62	80.0	90.0
**APT min**	0.533	0.301-0.765	0.782	1.45	33.3	100.0
**APT mean**	0.880	0.736-1.000	0.002*	2.79	73.3	100.0
**APT 10th percentile**	0.813	0.638-0.988	0.009*	1.84	66.7	100.0
**APT 25th percentile**	0.847	0.686-1.000	0.004*	1.89	80.0	90.0
**APT 50th percentile**	0.900	0.764-1.000	0.001*	1.94	93.3	80.0
**APT 75th percentile**	0.847	0.682-1.000	0.004*	2.35 or 3.21	93.3 or 73.3	70.0 or 90.0

**APT 90th percentile**	0.807	0.605-1.000	0.011*	2.88 or 3.50	93.3 or 73.3	70.0 or 90.0
**APT skewness**	0.507	0.242-0.771	0.956	-0.53	93.3	40.0
**APT kurtosis**	0.553	0.325-0.782	0.657	2.50	90.0	40.0
**TBF max**	0.533	0.302-0.764	0.782	65.45	26.7	100
**TBF min**	0.813	0.632-0.995	0.009*	7.35	70.0	93.3
**TBF mean**	0.660	0.431-0.889	0.183	30.50	50.0	93.3
**TBF 10th percentile**	0.727	0.518-0.935	0.059	13.21	80.0	73.3
**TBF 25th percentile**	0.680	0.463-0.897	0.134	16.84 or 20.62	80.0 or 60.0	53.3 or 73.3
**TBF 50th percentile**	0.653	0.425-0.881	0.202	29.74	50.0	86.7
**TBF 75th percentile**	0.633	0.401-0.866	0.267	31.78	60.0	73.3
**TBF 90th percentile**	0.600	0.364-0.836	0.405	45.26	40.0	86.7
**TBF skewness**	0.520	0.290-0.750	0.868	0.68	33.3	90.0
**TBF kurtosis**	0.767	0.558-0.975	0.027*	3.34	60.0	90.0
**ADC max**	0.640	0.414-0.866	0.244	1.88	60.0	80.0
**ADC min**	0.900	0.771-1.000	0.001*	0.51	100.0	73.3
**ADC mean**	0.673	0.460-0.887	0.149	0.86	100.0	46.7
**ADC 10th percentile**	0.827	0.664-0.990	0.007	0.80 or 0.94	90.0 or 70.0	66.7 or 86.7
**ADC 25th percentile**	0.773	0.588-0.959	0.023*	0.86	90.0	66.7
**ADC 50th percentile**	0.653	0.434-0.873	0.202	0.80	100.0	46.7
**ADC 75th percentile**	0.647	0.426-0.868	0.222	0.91	100.0	46.7
**ADC 90th percentile**	0.613	0.389-0.837	0.346	1.04	100.0	46.7
**ADC skewness**	0.700	0.486-0.914	0.096	0.50	80.0	60.0
**ADC kurtosis**	0.720	0.510-0.930	0.067	3.35	80.0	70.0

**APT50th+TBFmin+ ADCmin**	0.933	0.807-1.000	<0.001*			

Abbreviation: APT, amide proton transfer (%); TBF, tumor blood flow (mL/100g/min); ADC, apparent diffusion coefficient (10^{-3} mm^2 /s); max, maximum; min, minimum; AUC, area under the curve; CI, confidence interval. * *p* value <0.05
