# Supplementary figures and images for: A Combination of Amide Proton Transfer, Tumor Blood Flow, and Apparent Diffusion Coefficient Histogram Analysis Is Useful for Differentiating Malignant from Benign Intracranial Tumors in Young Patients: A Preliminary Study
Source: Diagnostics (Basel). 2024 Jun 12;14(12):1236. doi: 10.3390/diagnostics14121236 (PMC11202847; doi:10.3390/diagnostics14121236)

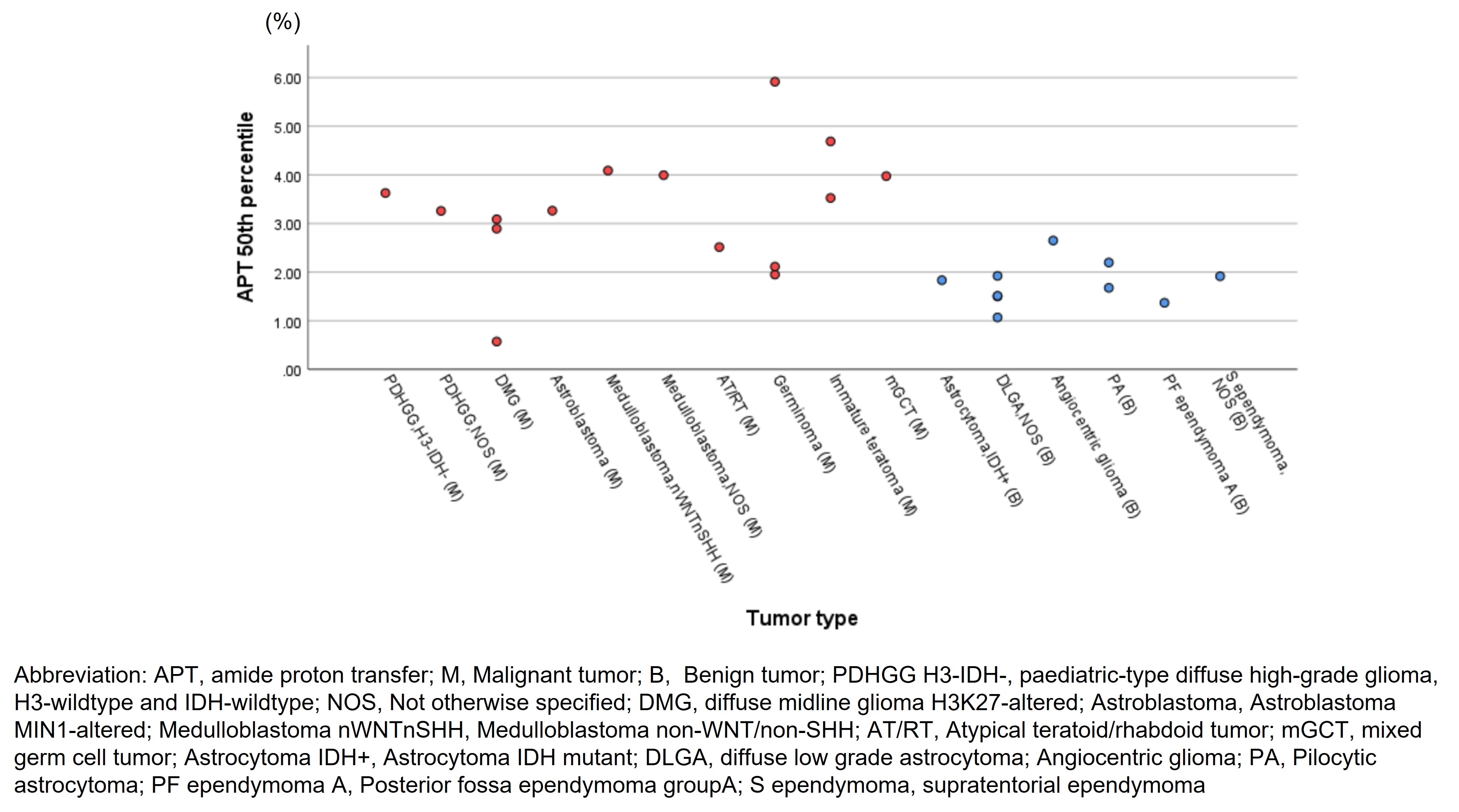

Supplement: Supplementary file 1 [file diagnostics-14-01236-s001.zip › Supplementary_Figure_S1a.png]

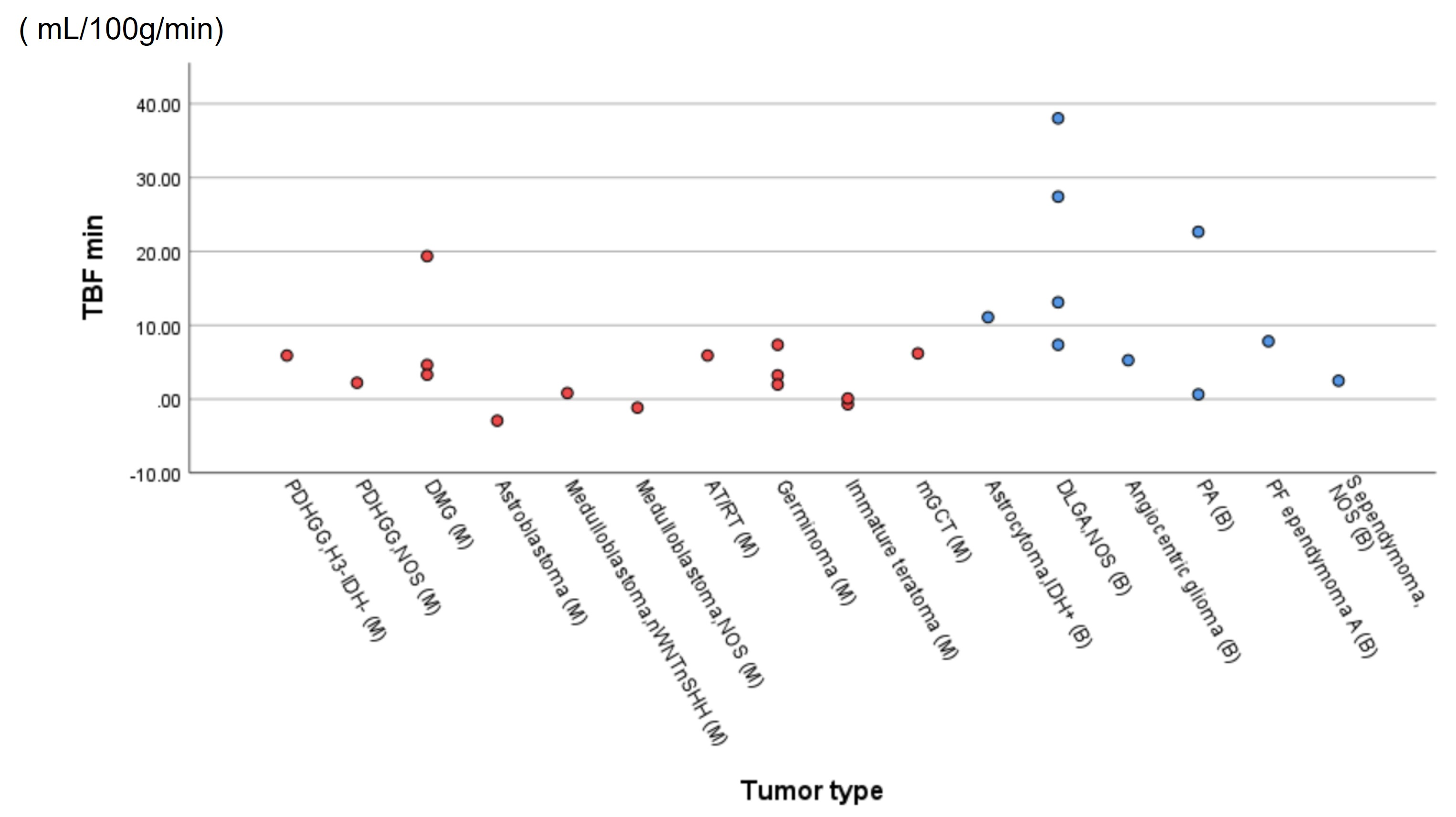

Supplement: Supplementary file 1 [file diagnostics-14-01236-s001.zip › Supplementary_Figure_S1b.png]

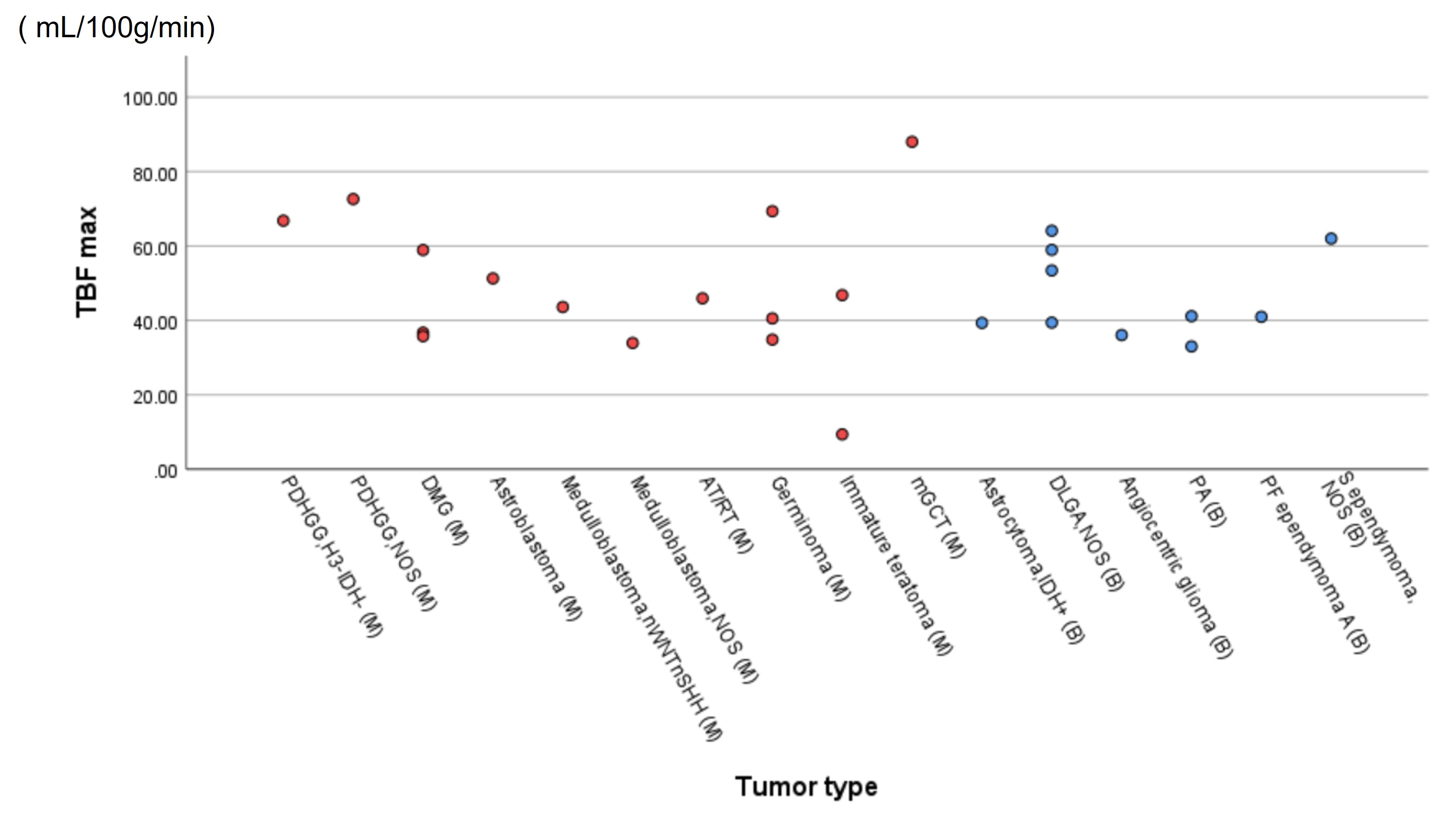

Supplement: Supplementary file 1 [file diagnostics-14-01236-s001.zip › Supplementary_Figure_S1c.png]

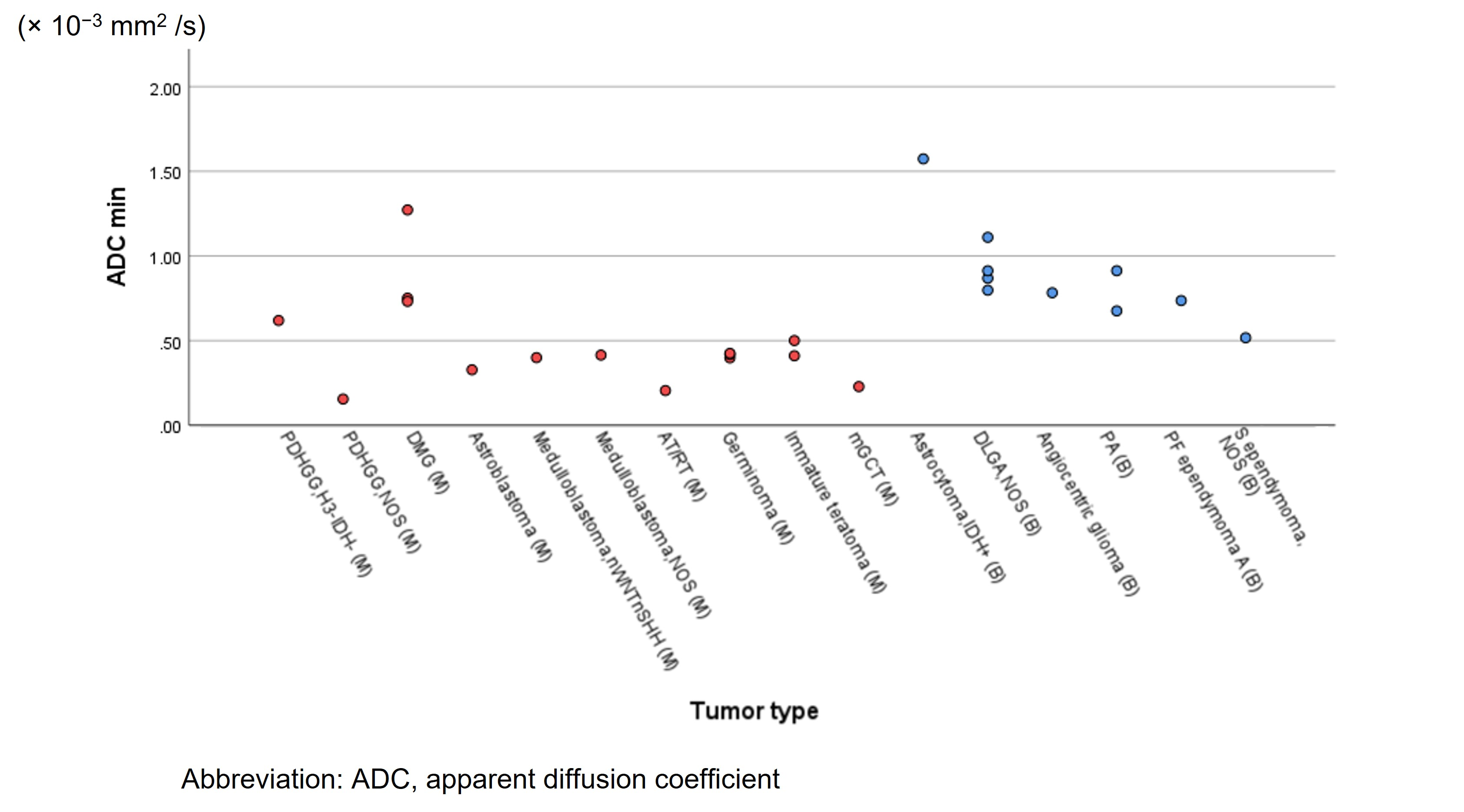

Supplement: Supplementary file 1 [file diagnostics-14-01236-s001.zip › Supplementary_Figure_S1d.png]
